# *Figuring Out* How Verb-Particle Constructions Are Understood During L1 and L2 Reading

**DOI:** 10.3389/fpsyg.2019.01733

**Published:** 2019-07-31

**Authors:** Mehrgol Tiv, Laura Gonnerman, Veronica Whitford, Deanna Friesen, Debra Jared, Debra Titone

**Affiliations:** ^1^Department of Psychology, McGill University, Montreal, QC, Canada; ^2^School of Communication Sciences and Disorders, McGill University, Montreal, QC, Canada; ^3^Department of Psychology, University of New Brunswick, Fredericton, NB, Canada; ^4^Faculty of Education, Applied Psychology, University of Western Ontario, London, ON, Canada; ^5^Department of Psychology, Brain & Mind Institute, University of Western Ontario, London, ON, Canada

**Keywords:** bilingualism, verb particle constructions, phrasal verbs, reading, eye tracking

## Abstract

The aim of this paper was to investigate first-language (L1) and second-language (L2) reading of verb particle constructions (VPCs) among English–French bilingual adults. VPCs, or phrasal verbs, are highly common collocations of a verb paired with a particle, such as *eat up* or *chew out*, that often convey a figurative meaning. VPCs vary in form (*eat up the candy* vs. *eat the candy up*) and in other factors, such as the semantic contribution of the constituent words to the overall meaning (semantic transparency) and frequency. Much like classic forms of idioms, VPCs are difficult for L2 users. Here, we present two experiments that use eye-tracking to discover factors that influence the ease with which VPCs are processed by bilingual readers. In Experiment 1, we compared L1 reading of adjacent vs. split VPCs, and then explored whether the general pattern was driven by item-level factors. L1 readers did not generally find adjacent VPCs (*eat up the candy)* easier to process than split VPCs (*eat the candy up)*; however, VPCs low in co-occurrence strength (i.e., low semantic transparency) and high in frequency were easiest to process in the adjacent form during first pass reading. In Experiment 2, we compared L2 reading of adjacent vs. split VPCs, and then explored whether the general pattern varied with item-level or participant-level factors. L2 readers generally allotted more second pass reading time to split vs. adjacent forms, and there was some evidence that this pattern was greater for L2 English readers who had less English experience. In contrast with L1 reading, there was no influence of item differences on L2 reading behavior. These data suggest that L1 readers may have lexicalized VPC representations that are directly retrieved during comprehension, whereas L2 readers are more likely to compositionally process VPCs given their more general preference for adjacent particles, as demonstrated by longer second pass reading time for all split items.

## Introduction

The comprehension of figurative language is a linguistically and cognitively demanding process that often requires inferences beyond the literal meaning. These cognitive demands are likely to be even greater for bilingual readers who vary continuously in terms of relative first-language (L1) and second-language (L2) experience. Much of the work on figurative language has analyzed the processing of idioms (e.g., *kick the bucket*), which are highly conventionalized multiword expressions with meanings that often transcend the semantics of their constituent words (Nunberg, 1978; Gibbs et al., 1989; Cacciari and Glucksberg, 1991; Titone and Connine, 1999; Abel, 2003; Libben and Titone, 2008; Titone and Libben, 2014; Titone et al., 2019). This body of research has focused on fixed expressions that tend to have words in fixed positions. However, not all idioms have as rigid a structure as items like *kick the bucket*. Our goal here was to investigate whether similar conclusions can be drawn about idiomatic structures that, as a class, inherently have more structural flexibility.

One form of idiomatic language that has great structural flexibility is verb particle constructions (VPCs), such as *chew out* or *finish up*, where flexibility in form is a defining feature of the construction. VPCs exist in some languages, such as English and German, but not in others, like French, Spanish, or Italian (Blais and Gonnerman, 2013). Of interest here is how bilinguals, who are fluent in one language that has VPCs (English) and one that does not (French), process these constructions within a sentence context in English. To address this issue, we present two experiments that investigate factors that influence the ease with which bilinguals naturally read VPCs varying in form in their L1 (Experiment 1) and L2 (Experiment 2).

### VPCs and Factors That Modulate Processing

Verb particle constructions, also known as phrasal verbs, are highly common collocations of a verb paired with a particle (adverb or preposition) to achieve a figurative meaning, such as *eat up, cut back*, and *chew out*. There are over 3000 VPCs in English (MacArthur and Atkins, 1974) that convey figurative or metaphorical information, much like classic forms of idioms. Furthermore, they are distinct from verbs that are simply followed by a preposition such as *eat up a tree*, where the eating is occurring in the branches of a tree (Fraser, 1976; Dagut and Laufer, 1985; Paulmann et al., 2015). Unlike the somewhat static nature of many idioms, VPCs are more syntactically moveable, in that their component words can be flexibly separated while still behaving as a single lexical unit (Booij, 1990; Johnson, 1991; Stiebels and Wunderlich, 1994; Jackendoff, 1995; Farrell, 2005; Gonnerman and Hayes, 2005). As in the classic literature on idiom processing, however, there is a longstanding and contentious debate as to whether VPCs are stored as single units in the lexicon (see Cappelle et al., 2010, for review). On the one hand, some VPCs can serve as morphological derivations, which allow, for example, the VPC, *fix up* to transform into the noun *a fixer-upper*. Many cite morphological derivation as evidence for the phrasal processing of VPCs as one unit (Los, 2004; Cappelle, 2005; Farrell, 2005). On the other hand, some researchers contend that because of the form flexibility of VPCs (e.g., *fix* can be separated from *up* by a noun phrase), they operate more as decomposable multi-word expressions (Chomsky, 1970; Di Sciullo and Williams, 1987).

Substantial research has been devoted to figuring out VPC processing. Most of this work has been limited to monolinguals. In what follows, we selectively review three modulatory factors of VPC processing among monolinguals.

#### Form

Most VPCs have both an adjacent form (*The girl will eat up the candy all at once*) and a split form (*The girl will eat the candy up all at once*) (Brehm and Goldrick, 2017). Therefore, one factor that may influence the ease with which a VPC is processed is the placement of the particle (i.e., adjacent vs. split from verb); however, VPC form is itself impacted by a variety of other syntactic, semantic, and discourse factors (Gries, 2003; Lohse et al., 2004). Past work has investigated whether monolingual readers process one form more easily than the other as a function of such linguistic constraints, like transparency and noun phrase length, and they generally find that adjacent forms facilitate comprehension when processing constraints are high (e.g., Gonnerman and Hayes, 2005). However, it remains an open question as to whether the form of VPCs generally affects the ease with which bilingual process them in a natural reading paradigm.

#### Semantic Transparency

A second factor that has been shown to influence the ease with which VPCs are processed by monolinguals is semantic transparency, that is, the extent to which the component words contribute to the overall meaning (e.g., Gonnerman and Hayes, 2005). For example, in transparent VPCs (e.g., *finish up*), the addition of the particle does not drastically alter the meaning of the verb, whereas in opaque VPCs (e.g., *chew out*) the meaning of the verb in the VPC is more dependent on the particle (Gries, 1999). Consequently, in opaque VPCs, the pairing of the verb and particle leads to a very different meaning than the verb alone. Whereas *chew* refers to a movement of the mouth, *chew out* refers to scolding. These items may be more lexicalized, functioning as a single unit (as is found with opaque classic idioms), and they tend to have more figurative meanings, as just demonstrated.

Gonnerman and Hayes (2005) asked monolingual participants to rate the similarity between the meaning of a verb and its corresponding VPC (e.g., how similar is *finish* to *finish up*?). The results provided evidence that skilled readers understand that VPCs vary in how much the overall meaning draws from the component verb meaning. Following the rating task, another group of participants performed a self-paced reading comprehension task. They found that semantic transparency significantly impacted reading time in general: sentences with opaque VPCs (e.g., *chew out*) were read more slowly than sentences containing transparent VPCs (e.g., *finish up*), suggesting that semantic input critically influences the time course of meaning retrieval. Moreover, participants read sentences with opaque VPCs (*chew out*) more slowly in the split form (e.g., *chew the children out*) than in the adjacent form (e.g., *chew out the children*). In contrast, sentences containing a transparent VPC (*finish up*) were read faster in the split form (e.g., *finish the task up*) than in the adjacent form (e.g., *finish up the task).*

One reason why opaque VPCs, such as *chew out*, are easier to process in the adjacent form than in the split form might relate to a lowered probability of a garden path misinterpretation. During sentence reading, a reader who reaches the verb “chew” in a split VPC may interpret it in the literal sense. Then, once the reader reaches “out” several words later, they may have to reparse the sentence to revise the initial interpretation. It is possible that opaque VPCs such as *chew out* are stored as a single lexical unit and retrieved directly in that form, which would explain why monolingual readers find them easier to process than the split form. Additionally, the adjacent form of opaque VPCs may have reduced processing demands compared to the split form (Hawkins, 1994, 2004). Verbs that drastically change meaning upon reading of the particle pose greater processing and working memory demands than verbs that remain generally consistent with the particle addition (Lohse et al., 2004).

#### Frequency

A third factor that may influence the ease with which VPCs are processed is frequency. The effects of frequency on single word reading are robust; more frequent words are processed more rapidly than less frequent words (Inhoff and Rayner, 1986; Rayner and Duffy, 1986). Others have extended these frequency effects to models of traditional idiom processing (e.g., Cronk et al., 1993). For example, in an eye-tracking study of classic idioms, Titone et al. (2019) assessed how both frequency and semantic transparency influenced early vs. late eye fixation measures. One of their main findings was that higher frequency idioms were read more rapidly on the first pass. However, if those highly frequent idioms were semantically transparent (such that the literal meaning was more viable), readers experienced interference, presumably because of the greater demands of an in-the-moment compositional analysis (see also Columbus et al., 2015 for similar findings with metaphor). It is possible that VPCs would behave differently because of their syntactic flexibility; however, no studies have directly assessed the role of frequency on VPC reading using a natural paradigm. Here, we investigated whether the frequency of adjacent VPCs biases readers to process the adjacent form more rapidly than the split form. Moreover, we probed whether any effects of frequency interact with the semantic transparency of the VPC.

Corpus data that records the frequency of VPC forms can provide some insight into differences between transparent and opaque VPCs in ease of processing of adjacent vs. split forms. Specifically, Lohse et al. (2004) found that opaque VPCs tended to appear more frequently in the adjacent form (*chew out the class*), whereas more transparent VPCs were equally likely to appear as adjacent (*finish up the meal*) or split (*finish the meal up*).

Despite the high frequency of VPCs in natural English language (MacArthur and Atkins, 1974), individuals may vary in their familiarity with this type of figurative language. Some research suggests that on the whole, bilinguals are not as familiar with VPC meanings as monolinguals, which seem to be among some of the most challenging L2 constructions to acquire (e.g., Neagu, 2007; Garnier and Schmitt, 2016). Thus, the central goal of the current study is to compare L1 and L2 bilingual readers of English on factors that affect the ease with which they read VPCs, a topic to which we now turn.

### VPC Processing in Bilinguals

Past research contends that comprehension of figurative language is highly challenging for L2 learners (Yorio, 1989; Laufer, 1997; Howarth, 1998; Heredia and Cieślicka, 2015). This difficulty may arise from less experience in one or both languages compared to monolinguals. As a result of splitting their time between languages, bilinguals, reading in their L2, may have reduced sensitivity to nuances in either one, which could preclude rapid meaning retrieval of conventionalized forms, especially when the conventionalized meaning is very different than the word-by-word meaning (Laufer, 1997; Matlock and Heredia, 2002; Hopp, 2007). Moreover, bilinguals reading VPCs in their L1 may be globally impacted by the totality of their bilingual experience and demonstrate similar reduced sensitivity to nuances in each language. We aim to address both questions in the present work.

#### Bilingual VPC Production

Several studies have found that L2 users generally prefer single word synonyms (e.g., “scold”) over VPCs (e.g., “chew out”), especially when the VPC is less semantically transparent (e.g., Dagut and Laufer, 1985). This conclusion may depend on the language pair of the bilingual or the complexity of the L2. For example, studies on Hebrew-English bilinguals, where VPCs are not present in Hebrew but are present in English, have revealed L2 VPC avoidance behavior (Dagut and Laufer, 1985; Laufer and Eliasson, 1993). In contrast, Dutch-English bilinguals who have VPCs in both languages do not always avoid VPCs (Hulstijn and Marchena, 1989; Laufer and Eliasson, 1993; for a full discussion, see Liao and Fukuya, 2004). Given other work suggesting that this pattern changes as a function of proficiency in the L2 (Liao and Fukuya, 2004; Blais and Gonnerman, 2013), many conclude that L2 VPC avoidance is in part due to the constructional overlap between L1 and L2 (e.g., Kellerman, 1977). However, others contend that avoidance may rely less on how close or distant the language pairs may be and instead arises due to the L2 user’s mere *perception* that a multi-word phrase is more difficult to produce than a single word (Sjoholm, 1995).

#### Bilingual VPC Comprehension

Although much is known about VPC production in bilinguals, there is little research on how bilinguals comprehend VPCs. As pointed out by Blais and Gonnerman (2013), it is crucial to address VPC comprehension for a number of reasons. These include the fact that receptive language processing is a critical precursor for language production, and it often predicts productive language use in L1 and L2 (e.g., Benedict, 1977; Ringbom, 1992).

One of the few initial VPC comprehension studies investigated L2 reading of literal verb preposition (e.g., *eat up the tree*) vs. idiomatic VPCs (e.g., *eat up the candy*) in bilinguals who acquired the L2 early in life or later in life (Matlock and Heredia, 2002). The researchers found that whereas early bilinguals read the idiomatic VPC more rapidly, late bilinguals were much faster at reading the literal verb preposition construction. This was taken as evidence that early bilinguals benefit from rapid access to idiomatic meanings, much like L1 or monolingual users, which the late bilinguals do not possess (see also Paulmann et al., 2015 for similar results with ERPs). This early work used a self-paced reading paradigm (which may lack ecological validity) and did not focus on individual differences in bilingual experience, such as L2 usage or exposure.

More recently, Schunack (2016) found that German–Norwegian bilinguals demonstrated a preference for adjacent VPCs in an acceptability judgment task. Important, however, is that both German and Norwegian contain VPCs, though German has more limited form flexibility than Norwegian. In contrast, other common language pairings (e.g., French and English in Canada) may not both have VPCs. Thus, when examining VPC processing in bilinguals, it may be important to consider the specific language pairing because L1–L2 similarity also influences preferences of other figurative language (Laufer, 2000).

Blais and Gonnerman (2013) specifically focused on the processing of VPCs by French–English bilingual adults, which were the two languages of the bilinguals tested in the current study. They used the same items from Gonnerman and Hayes (2005). First, the authors compared French–English bilingual ratings of semantic similarity between the verb and VPC to English monolingual ratings. They found that although bilinguals were sensitive to semantic gradations in similarity between the VPC and the component verb (even in their L2), they never fully reached monolingual-like performance in the explicit judgment task despite showing improvement with increased proficiency. Specifically, the explicit L2 semantic judgments overlapped more closely with monolingual judgments for transparent VPCs, but less so for opaque VPCs. In addition, the authors measured VPC processing with an implicit masked priming task and found that response times were facilitated for semantically transparent but not opaque items. From this work, we know that bilinguals are sensitive to the measures that have been found to modulate monolingual form preference (i.e., semantic transparency); however, this study did not directly investigate whether bilingual reading was affected by other factors, such as frequency, or whether these factors impacted form preference.

Recently, Herbay et al. (2018) investigated a variety of item and participant factors that could potentially influence French–English bilinguals’ reading of English (L2) VPCs. Using a self-paced reading task, participants read the same VPC items from Gonnerman and Hayes (2005) and Blais and Gonnerman (2013). The authors manipulated both item-level properties (i.e., particle position, noun phrase length, semantic transparency) and participant-level properties (i.e., working memory capacity, VPC knowledge). Interestingly, these five factors significantly interacted; participants with good VPC knowledge and high working memory displayed a processing preference for adjacent particles when the VPC was semantically opaque and when the direct object noun phrase was long. In other words, L2 readers preferred the adjacent form when processing demands were high (i.e., low semantic transparency and long direct object). For example, more proficient bilinguals preferred the adjacent form to the split form of “*She will chew her facetious friend from school out*,” as demonstrated by shorter reading times. Thus, some L2 readers demonstrated sensitivity to similar factors that are known to impact monolingual reading of VPCs, such as particle placement and transparency.

## The Present Study

While these studies have revealed the cognitive factors involved in VPC processing, it is unknown whether the findings would extend to a more naturalistic reading situation. The relevant work on bilingual VPC reading has been limited to self-paced reading (along with some ERP work), which, while informative, may not provide a full picture of the processing difficulties that readers face. Thus, we investigated how bilingual adults naturally read VPCs that vary in form (adjacent vs. split) while their eye movements were recorded. We did so in both L1 and L2 bilingual groups, given that bilingualism may confer global changes in reading behavior that manifest differently during L1 vs. L2 reading. From this general question, we zoom in on whether the relative contributions of semantic transparency vs. frequency render greater ease of processing for one VPC form over the other, as demonstrated by shorter fixation times. The answer to this question may shed light on whether some VPCs are easier to directly retrieve (i.e., are lexicalized and function as a single unit) compared to others. We will also examine whether individual differences in L2 English usage affect L2 VPC reading, through changes in fixation times.

Specifically, we pursued three experimental questions:

(1) Do L1 and L2 readers process adjacent VPCs more easily than split VPCs? If adjacent particle placement is preferred, fixation times should be shorter on adjacent VPCs than split VPCs.
(2) Do the verb-particle semantic relationship and frequency jointly modulate early and late measures of L1 and L2 reading of sentences containing VPCs?
(3) With respect to L2 VPC reading, do individual differences in bilingual language experience (i.e., L2 English usage) modulate how these linguistic factors impact reading?

In Experiment 1, we address questions 1 and 2 for L1 readers of English (L2 French). In Experiment 2, we address all three questions for L2 readers of English (L1 French). Importantly, both groups only have VPCs in one of their languages, English.

## Methods Common to Experiments 1 and 2

### Materials

Seventy-eight VPCs were taken from Blais and Gonnerman (2013). These included items that were low, medium, and high on semantic similarity between the VPC and the verb (e.g., “look up” vs. “look”), as demonstrated in past work (Gonnerman and Hayes, 2005; Blais and Gonnerman, 2013). The VPCs also ranged on other aspects (e.g., verb heaviness, noun phrase complexity) that were not directly relevant to the current study. Furthermore, the specific particles (e.g., *up*) were distributed equally among the three semantic similarity groups.

Each of the VPCs was embedded into the middle of a sentence frame. All sentence frames began with a simple noun phrase + “will” at the start, and had a noun, prepositional, or adverbial phrase appended to the end. Two versions of each sentence frame were created, one containing the adjacent form of the VPC, and one containing the split form. The versions only differed in placement of the particle. Otherwise, all other aspects (including length) were identical. In the adjacent condition the particle appeared immediately after the verb (e.g., *The girl will eat up the candy all at once*), whereas in the split condition, a noun phrase appeared between the verb and the particle (*The girl will eat the candy up all at once*). The sentences were counterbalanced, such that each participant only saw one version of each sentence frame (e.g., “eat up” as adjacent *or* split). Each participant viewed all 78 items, which consisted of an equal number of sentences with adjacent vs. split VPCs.

### VPC Linguistic Properties

We assessed two linguistic properties of each VPC: frequency and verb-particle co-occurrence strength. We believe co-occurrence strength to be another way to measure the semantic relationship between the verb and the particle, one that can be derived from objective corpus data. In contrast, past work including Blais and Gonnerman (2013) assessed the semantic dependency of the VPC through human ratings, which may be more subjective than corpus data. Corpus measures were used in place of traditional human ratings principally because these ratings vary among monolinguals and bilinguals. Specifically, past work demonstrates that though L2 ratings for VPCs correlate with monolingual ratings, there are still significant differences between the two groups, which can be explained by individual differences in L2 proficiency (Blais and Gonnerman, 2013). Thus, we aimed to avoid rating measures that were tagged to a specific population (e.g., monolinguals vs. bilinguals), and used corpus measures as a benchmark value of overall frequency and semantic co-occurrence across the general linguistic environment. Frequency values were obtained for each VPC using the Corpus of Contemporary American English (COCA; Davies, 2008). COCA is a widely used corpus of written American English and provides robust information on the frequency of words and collocations. In all analyses, COCA frequency of the adjacent verb + particle form (e.g., *eat up)* served as the metric for frequency, as it reflected how entrenched each canonical VPC was.

Verb particle constructions’ co-occurrence strength was indexed using Latent Semantic Analysis (LSA) Cosine (Laham, 1998)^1^. LSA is also a widely used corpus-based tool for assessing semantic relatedness among linguistic materials (Landauer and Dumais, 1997; Landauer et al., 1998; Kintsch, 2000). LSA utilizes the cosine of vectors between specific words and the general contexts in which they occur, to find meaning similarity among words and phrases in a manner that mimics human judgments of linguistic similarity (Laham, 1998). However, unlike human judgments, it will not be conflated with individual familiarity or frequency. In deciding how to formulate the LSA web interface search, there were multiple options (e.g., verb to VPC overlap, particle to VPC overlap, verb to particle overlap). We decided to gather cosine values of overlapping verb and particle context (i.e., “bump” to “off”). Theoretically, this search made the most sense given our aim of weighing the semantic contribution of each constituent part of the VPC. Furthermore, of the three search possibilities, the verb-particle overlap had the greatest correlation with past human ratings from bilingual adults (taken from Blais and Gonnerman, 2013). The specific parameters we used in this search were term-to-term matrix comparisons of general reading up to the first year of college with maximal factors. A more positive score indicates greater co-occurrence strength between the verb and the particle, whereas a more negative score suggests less co-occurrence strength. In other words, high co-occurring pairs (e.g., *bump off)* tend to exist in similar semantic spaces, suggesting that the VPC meaning is transparent or not solely dependent on the particle. However, less co-occurring pairs (e.g., *chew out)* exist in separate semantic spaces, thus implying that the construction may be opaque, figurative, and more lexicalized when the verb and particle co-occur. This measure may link to past measures of semantic transparency.

**FIGURE 1:**
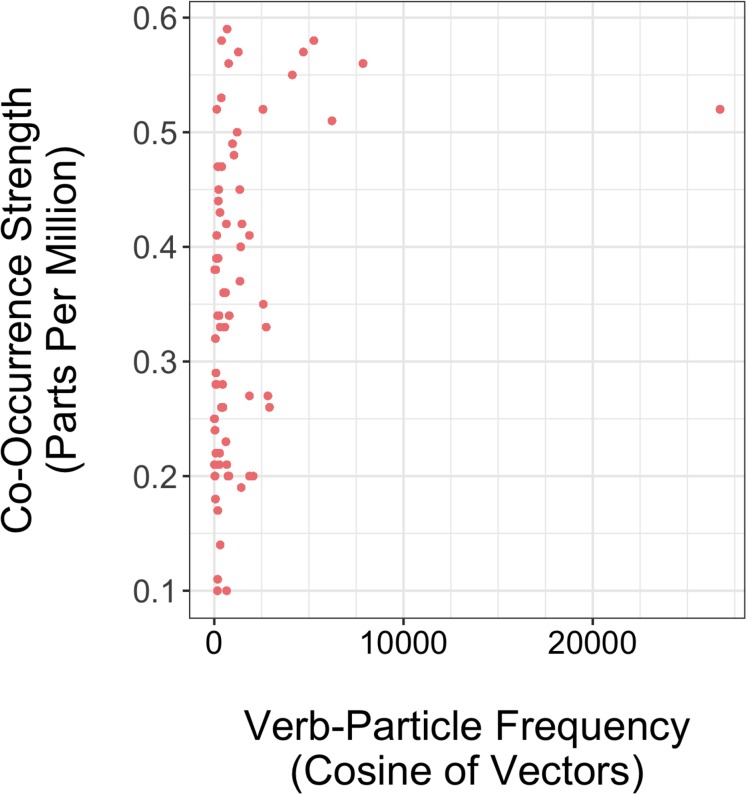
Scatterplot depicting raw values for Co-Occurrence Strength and Verb-Particle Frequency for 78 VPC.

Figure 1 depicts the distribution of items across both linguistic properties. Here, one may observe a trend for high frequency VPCs to also appear high on co-occurrence strength. Indeed, these values are somewhat correlated (0.31), but the correlation substantially increases with the exclusion of the potential outlier item on frequency, “find out” (0.41). For this reason, all items were included in the analysis, and the implications of including this item will be discussed in Experiment 2.

### Procedures

Participants first completed a language history questionnaire (Li et al., 2014), which asked questions regarding time spent speaking, reading, writing, and listening in each of their languages. Participants whose L1 was not French or English, or those who spent more than 20% of the time in a third-language (L3), were excluded from analysis. Participant characteristics are provided in Table 1.

**TABLE 1:** Descriptive statistics for participants.

	**Mean**	***SD***	**Minimum**	**Maximum**
**English L1**				
**Experiment 1**				
Age (in Years)	20.89	3.82	17	30
L2 Usage (%)	12.51	17.35	0	50
L2 AoA (Age in years)	7.20	4.09	1	23
**French L1**				
**Experiment 2**				
Age (in Years)	20.95	2.5	19	29
L2 Usage (%)	56.96	23.55	20	90
L2 AoA (Age in years)	6.90	4.35	0	19

*AoA, age of acquisition.*

Participants read sentences presented as single lines on the computer screen while their right eye movements were recorded with an EyeLink 1000 eye-tracker (sampling rate = 1 kHz, SR Research, Mississauga, ON, Canada). At the start of the experiment (and after each break) a 5-point star calibration was used. During the experiment, sentences were presented on a black screen in size 10 Monaco yellow font on a 20-inch CRT monitor, positioned 71 cm away from participants. Participants pressed a button on a controller pad when they completed reading each sentence. The experiment was a part of a larger study consisting of 292 total trials and 20 yes/no comprehension questions looking at L1 and L2 reading. In total, the experiment took two hours, and participants were given the choice to take brief pauses in addition to the three scheduled breaks. This study was carried out in accordance with the recommendations of McGill University’s Research Ethics Board (REB), which also approved the protocol. All subjects gave written informed consent in accordance with the Declaration of Helsinki.

### Comprehension Performance

Participants responded to 20 yes/no comprehension questions on filler sentences. The average accuracy for L1 English participants (Experiment 1) was 90.3%, and the average accuracy for L2 English participants (Experiment 2) was 89.6%. Across the experiments, five participants’ responses to comprehension questions were excluded due to technical issues with the response button box. These results suggest that both groups were attentive throughout the experimental session.

### Eye-Tracking Measures

We removed fixations less than 80 ms, trials whose target regions were not fixated, and blinks. Two regions were of interest: the VPC region (including the verb, particle, and noun phrase, for example, *eat up the candy/eat the candy up*) and the post-VPC spillover region (e.g., *all at once).* The full VPC region was selected in order to compare reading across the adjacent and split conditions. Given that the two conditions of each item were composed of the same words but in a different order, the region lengths are the same (mean length = 19.3 characters; standard deviation of length = 2.3). The post-VPC spillover region was selected to capture any residual, spillover reading effects and was identical across conditions (mean length = 11.5 characters; standard deviation of length = 3.6).

We calculated gaze duration for the VPC region and additional reading time in the post-VPC spillover region. Gaze duration, which reflects the initial stages of lexical access, measures fixation durations starting when the eye first lands on a region until it moves out of the region (i.e., first pass reading). Next, we calculated second pass reading time for the same region. Second pass reading time was calculated by subtracting gaze duration from total reading time, or the total amount of time spent in a region. In turn, second pass reading time reflects *additional* processing of a region, particularly with respect to sentence- or discourse-level factors (for a review of eye-tracking measures, see Titone et al., 2016). Of note, in cases where participants only fixated the regions of interest on the first pass, the second pass time would be 0, and this would be included in the analysis. In effect, when the region was fixated on the first pass, we are examining the amount of time readers initially spend in that region, as well as the amount of *additional* time readers spend in rereading that region. We then used these two reading measures as predictors in the first set of analyses.

We approached the data analysis in this manner for two reasons. First, to reduce the inherent redundancy between gaze duration and total reading time measures, where the latter normally subsumes the former. Second, to reduce the number of core statistical models necessary to draw conclusions about early versus late processing across the VPC and post-VPC regions (see von der Malsburg and Angele, 2017 for additional discussion of these issues). Having a pure measure of rereading time, as measured by second pass reading time, is important in this investigation because we are interested in the *additional processing time* that potentially difficult expressions incur on comprehension. However, past work has demonstrated that including a large proportion of zeros distorts the residuals of the models and may violate the normality assumption for linear mixed effects regressions, even if the reading times are log-transformed. The loss of power that accompanies non-normal residual distribution may contribute to increased Type II errors, that is, failing to detect an effect that is present (Vasishth et al., 2012). Thus, we checked and report the residual distributions of each model in what follows.

### Availability of Data

The de-identified data relevant to the conclusions of this paper and the R script used to generate these conclusions are publicly available on the Open Science Foundation project^2^. Supplementary Materials can also be found here.

## Experiment 1: L1 Reading of VPC

Experiment 1 investigated bilinguals’ overall preferences for particle placement (adjacent vs. split) when reading VPCs in their L1 (English), which has VPCs. Fifty-six English–French bilingual university students with normal or corrected vision and no self-reported history of speech, hearing, learning, reading, neurological, or psychiatric disorders, participated for course credit or monetary compensation ($10/h). Participants were recruited from both McGill University and the University of Western Ontario. They reported speaking French (L2) 0–50% of the time. Their mean L2 usage was 12.51%, and their mean L2 age of acquisition (AoA) was 7.2 years. Full participant descriptive statistics are available in Table 1.

The data were analyzed using linear mixed-effects models (LMMs) in R (R Development Core Team, 2015; version 3.2.3) via the lme4 package (Bates et al., 2015). For all models, the eye movement measures were log transformed to normalize their distribution (added 1 to all values before log-transformation because of presence of zeroes), and all categorical variables were deviation coded (+ 0.5, −0.5). We conducted two sets of analyses for L1 reading of VPC: Analysis 1 assessed participants’ general preference for particle placement, and Analysis 2 explored whether preference for particle placement varied as a function of VPC frequency and co-occurrence strength. Across all models, we evaluated significance using Satterthwaite approximations, implemented in the *lmerTest* package (Kuznetsova et al., 2015).

### Analysis 1: Does Form Modulate L1 VPC Reading?

Following Barr et al. (2013), we computed an LMM with maximal random effects by subjects and by items for the core interaction between reading measure (gaze duration vs. second pass reading time) × VP position (adjacent vs. split) × region (VPC vs. post-VPC spillover region). The random interaction slope was dropped due to lack of convergence.

The goal of this analysis was to determine whether L1 readers demonstrate overall preference for the adjacent or split form of VPCs with their eye movements. If they do demonstrate a general preference, we expect faster reading times for the adjacent form, which decreases processing demands compared to the split form (Hawkins, 1994, 2004).

**FIGURE 2:**
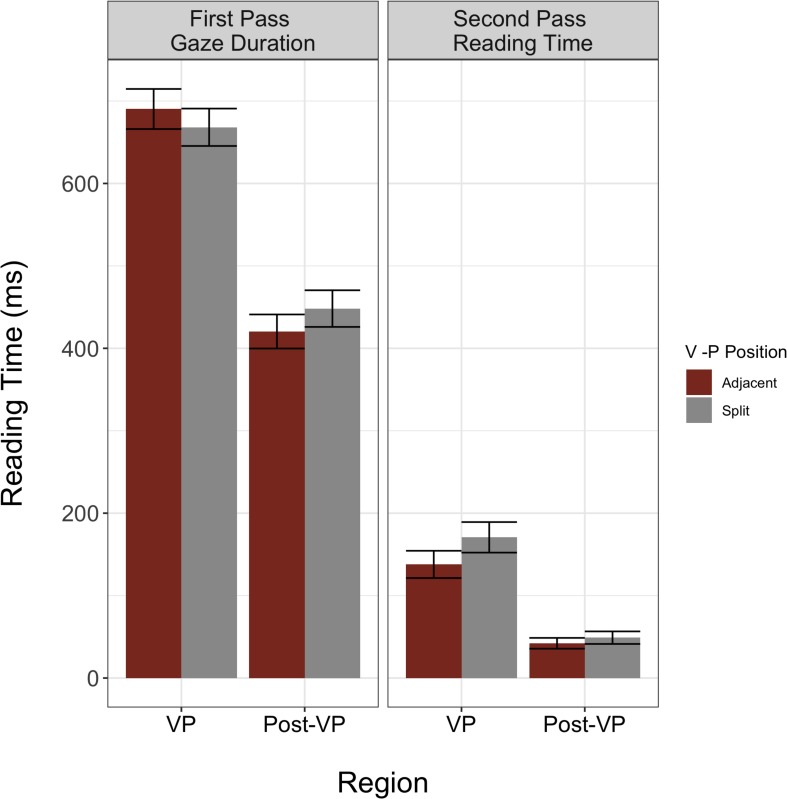
L1 raw reading times for core model (Region × Measure × VP Position). Model indicates a significant interaction between measure and region. Error bars indicate plus/minus one standard error of the mean.

Using this procedure, we found significant main effects of reading measure (β = −4.87, *SE* = 0.13, *p* < 0.001) and region (β = −0.54, *SE* = 0.07, *p* < 0.001). These data suggest that generally, second pass reading time is shorter than first pass reading, and that reading of the VPC region is longer than the post-VPC region (Figure 2). Furthermore, we found a significant interaction between measure and region (β = −0.58, *SE* = 0.07, *p* < 0.001). To understand the nature of this interaction, we computed the difference in reading time at the VPC and post-VPC spillover regions for first and second pass reading. Whereas the decrease in reading time from the VPC to the post-VPC spillover region is numerically larger during first pass reading (240 ms vs. 104 ms), the percent decrease in reading time from VPC to post-VPC spillover region was greater during second pass (68.5%) than first pass reading (34.7%). However, given that neither of these variables interacted with VP position, we did not detect a general difference in adjacent vs. split particle placement preference among these L1 readers of English. The full model outputs for Analysis 1 are available in Table 2. In checking the model residuals, we found that these values deviated from normality. Moreover, the non-normal distribution was similar when the model included trials where the post-VPC spillover region was not fixated.

**TABLE 2:** Experiment 1, Analysis 1 core model outputs.

**Fixed effects**	***b***	***SE***	***t-value***	***p-value***
(Intercept)	6.13	0.04	161.11	< 0.001^*^
Measure	–4.87	0.12	–39.00	< 0.001^*^
Region	–0.53	0.07	–7.66	< 0.001^*^
VP Position	–0.01	0.05	–0.21	0.83
Measure × Region	–0.58	0.07	–8.78	< 0.001^*^
Measure × VP Position	–0.09	0.07	–1.42	0.15
Region × VP Position	–0.10	0.09	–1.12	0.26
Measure × Region × VP Position	0.24	0.13	1.83	0.07

**Random effects**				**Variance**

Item	(Intercept)			0.00
	Region			0.02
	Measure			0.23
	VP Position			0.03
Participant	(Intercept)			0.05
	Region			0.13
	Measure			0.64
	VP Position			0.02

^*^p < 0.05.

lmer[Log RT ∼ Measure^*^Region^*^Position + (1 + Region + Measure + Position| Subject) + (1 + Region + Measure + Position| item), vp_L1].

### Analysis 2: Do Co-occurrence Strength and Frequency Modulate L1 VPC Reading?

In Analysis 1, we did not find evidence that L1 readers have an overall preference for particle placement; however, it is quite plausible that characteristics specific to certain items, such as frequency or co-occurrence strength between the verb and particle, impact particle preference. Given past findings, we expect both frequency and semantic co-occurrence strength to impact reading patterns. Whether these two linguistic dimensions exert independent or interactional effects will be determined by this second analysis.

We computed a series of LMMs with random intercepts by subjects and items for the item-specific models. Here, we fit an interaction between VP position (adjacent vs. split) × scaled verb particle frequency × scaled co-occurrence strength. This interaction was tested for each reading measure and region individually, thus resulting in four models (gaze duration at the VPC, gaze duration at the Post-VPC spillover, second pass reading time at the VPC, and second pass reading time at the Post-VPC spillover). Of these models, we only report significant interactions between frequency or co-occurrence strength and VP position, though full model outputs are provided in Table 3.

**TABLE 3A:** Experiment 1, Analysis 2 item-specific model outputs at the VPC region.

**Gaze duration**				
**Fixed effects**	***b***	***SE***	***t-value***	***p-value***
(Intercept)	6.36	0.04	153.35	< 0.001^*^
VP Position	0.00	0.03	0.06	0.95
Frequency (scaled)	–0.15	0.11	–1.41	0.16
Co-occurrence Strength (scaled)	0.00	0.04	–0.24	0.81
VP Position × Frequency (scaled)	–0.36	0.17	–2.17	0.03^*^
VP Position × Co-occurrence Strength (scaled)	0.12	0.06	2.04	0.04^*^
Frequency (scaled) × Co-occurrence Strength (scaled)	0.21	0.16	1.29	0.20
VP Position × Frequency (scaled) × Co-occurrence Strength (scaled)	0.55	0.25	2.16	0.03^*^

**Random effects**	**Variance**			

Item (Intercept)	0.01			
Participant (Intercept)	0.07			

**Second pass reading time**				
**Fixed Effects**	***b***	***SE***	***t-value***	***p-value***

(Intercept)	1.79	0.17	10.73	< 0.001^*^
VP Position	–0.13	0.14	–0.97	0.33
Frequency (scaled)	0.00	0.49	0.01	0.99
Co-occurrence Strength (scaled)	0.26	0.17	1.60	0.12
VP Position × Frequency (scaled)	0.09	0.75	0.12	0.91
VP Position × Co-occurrence Strength (scaled)	0.10	0.25	0.38	0.70
Frequency (scaled) × Co-occurrence Strength (scaled)	0.30	0.75	0.40	0.69
VP Position × Frequency (scaled) × Co-occurrence Strength (scaled)	–0.51	1.11	–0.46	0.65

**Random effects**	**Variance**			

Item (Intercept)	0.18			
Participant (Intercept)	1.08			

^*^p < 0.05.

Gaze Duration: lmer[Log RT ∼ position^*^scaled frequency^*^scaled co-occurrence + (1 | Subject) + (1 | item), L1_VP_GD]. Second Pass: lmer[Log RT ∼ position^*^scaled frequency^*^scaled co-occurrence + (1 | Subject) + (1 | item), L1_VP_SP].

**TABLE 3B:** Experiment 1, Analysis 2 item-specific model outputs at the post-VPC region.

**Gaze duration**				
**Fixed effects**	***b***	***SE***	***t-value***	***p-value***
(Intercept)	5.86	0.05	108.55	< 0.001^*^
VP Position	–0.03	0.03	–1.07	0.28
Frequency (scaled)	0.17	0.18	0.98	0.33
Co-occurrence Strength (scaled)	–0.11	0.06	–1.79	0.08
VP Position × Frequency (scaled)	0.19	0.16	1.25	0.21
VP Position × Co-occurrence Strength (scaled)	–0.08	0.05	–1.60	0.11
Frequency (scaled) × Co-occurrence Strength (scaled)	–0.22	0.27	–0.85	0.40
VP Position × Frequency (scaled) × Co-occurrence Strength (scaled)	–0.38	0.24	–1.61	0.11

**Random effects**	**Variance**			

Item (Intercept)	0.04			
Participant (Intercept)	0.10			

**Second pass reading time**				
**Fixed effects**	***b***	***SE***	***t-value***	***p-value***

(Intercept)	0.63	0.09	7.01	< 0.001^*^
VP Position	–0.07	0.10	–0.73	0.47
Frequency (scaled)	–0.33	0.32	–1.05	0.30
Co-occurrence Strength (scaled)	0.22	0.11	2.09	0.04^*^
VP Position × Frequency (scaled)	–0.21	0.52	–0.40	0.69
VP Position × Co-occurrence Strength (scaled)	–0.09	0.18	–0.47	0.64
Frequency (scaled) × Co-occurrence Strength (scaled)	0.53	0.48	1.11	0.27
VP Position × Frequency (scaled) × Co-occurrence Strength (scaled)	0.62	0.79	0.78	0.44

**Random Effects**	**Variance**			

Item (Intercept)	0.06			
Participant (Intercept)	0.24			

^*^p < 0.05.

Gaze Duration: lmer[Log RT ∼ position^*^scaled frequency^*^scaled co-occurrence + (1 | Subject) + (1 | item), L1_Spill_GD]. Second Pass: lmer[Log RT ∼ position^*^scaled frequency^*^scaled co-occurrence + (1 | Subject) + (1 | item), L1_Spill_SP].

**FIGURE 3:**
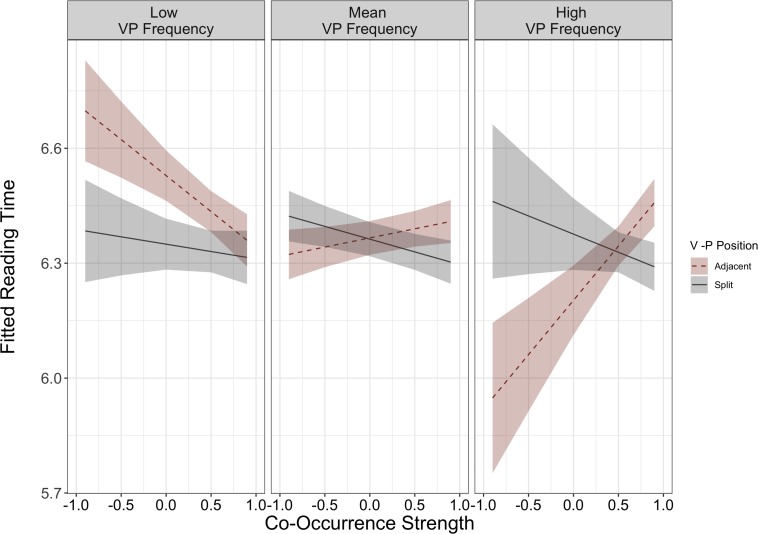
Model fitted L1 reading time for item-specific model (VP Position × Frequency × Co-Occurrence Strength) for Gaze Duration at the VP region. Mean VP frequency presented in middle panel. Low VP frequency illustrates one standard deviation below the mean, and high VP frequency illustrates one standard deviation above the mean.

There were two, two-way interactions involving VP position for gaze duration at the VPC region: VP position × frequency (β = −0.36, *SE* = 0.17, *p* = 0.03) and VP position × co-occurrence strength (β = 0.11, *SE* = 0.06, *p* = 0.04). These interactions were qualified by a significant higher level, three-way interaction between VP position × frequency × co-occurrence strength for gaze duration at the VPC region (β = 0.54, *SE* = 0.25, *p* = 0.03). To understand this interaction, we conducted two *post hoc* LMMs on subsets of only adjacent VPCs and only split VPCs. These models indicated that the frequency × co-occurrence interaction was present among the adjacent items, and not the split items (adjacent subset: β = 0.55, *SE* = 0.23, *p* = 0.02; split subset: β = −0.06, *SE* = 0.22, *p* = 0.77). This interaction is illustrated in Figure 3, and indicates that readers display shorter reading times for highly frequent and low co-occurrence strength VPCs in the adjacent form (e.g., *cut back*). This preference for the adjacent form was not observed for opaque VPCs that are low in frequency, and in fact, Figure 3 illustrates that low frequency, opaque items are read the most slowly in the adjacent form. Importantly, the interaction between frequency and semantic co-occurrence strength was not observed in any of the other three models (second pass reading time at the VPC, second pass reading time at the post-VPC spillover region, or first pass reading time at the post-VPC spillover region). Again, the model residuals for this interaction slightly deviated from normality and should be interpreted with that in mind.

## Experiment 1 Discussion

To summarize, Experiment 1 examined L1 reading of VPCs among English-French bilingual adults. In Analysis 1, we investigated whether adjacent or split forms of the VPC modulate L1 reading as measured by changes in fixation times. Here, we failed to detect a general processing difference for adjacent vs. split particle placement across all items. In other words, reading times did not change when comparing all adjacent vs. split particle placement items. This initial pattern of results contributed to the next analysis where we added VPC linguistic properties to the models.

In Analysis 2, we explored whether continuous differences in co-occurrence strength and frequency affected L1 reading of adjacent vs. split VPCs. Here, there was a significant interaction involving both measures during gaze duration at the VPC region: When L1 readers first read the VPC region, they demonstrate a preference for the adjacent form (i.e., faster reading times) only when the VPC was low in semantic co-occurrence strength (opaque) and high in frequency (e.g., *cut back*). The preference for adjacent forms was not found if the low semantic co-occurrence strength items were less frequent (e.g., *chew out*). In other words, highly frequent and opaque items (i.e., the items that were most likely to be lexicalized) were retrieved most quickly in the adjacent form. This pattern of results mirrors past findings from monolingual readers, who prefer opaque VPCs in the adjacent form during self-paced reading (Gonnerman and Hayes, 2005). Not only did that work use a different task, but it also did not examine how frequency modulates this relationship, whereas our results indicate that frequency is critical in predicting whether a semantically opaque VPC is preferred in the adjacent or split form. This suggests that the interplay of frequency and semantic co-occurrence strength is interactional, and not independent, such that the frequency of a phrase in the linguistic environment modulates the effects that semantic co-occurrence exerts. One could understand this pattern of results as evidence for a preferred direct meaning retrieval mechanism over word-by-word composition. When the VPC optimally matches what is likely stored in the lexicon (i.e., opaque, adjacent form) the meaning is retrieved quickly (Titone et al., 2019). However, when this mapping is less frequent or familiar, retrieval is less rapid, which aligns with recent work demonstrating that more figuratively-dominant idioms are read more quickly than idioms with more balanced figurative-literal meanings (Milburn and Warren, 2019). Similarly, when the VPC is semantically decomposable or transparent, reading time increases because the meaning is being constructed on the spot. Furthermore, this interaction between these linguistic properties and the preferred form was not observed in the spillover region or during second pass reading. Thus, these data suggest that frequency and semantic information can be accessed at very early stages of reading, and they can be accessed at the same time.

However, note that for both significant effects, the model residuals were noticeably non-normal. The loss of power that accompanies non-normal residual distribution may contribute to increased Type II errors, that is, failing to detect an effect that is present (Vasishth et al., 2012). As a result, it is possible that there is more to the results than what is described here. Next, we turn to how the same VPCs are processed by L2 readers of English.

## Experiment 2: L2 Reading of VPC

Experiment 2 investigated bilingual preferences for particle placement when reading VPCs in their L2 (English). Twenty-seven French-English bilingual university students with normal or corrected vision and no self-reported history of speech, hearing, learning, reading, neurological, or psychiatric problems, participated for course credit or monetary compensation ($10/h). The smaller sample size compared to Experiment 1 was because these participants were all recruited from McGill University, whereas the participants in Experiment 1 were recruited from a combination of McGill University and the University of Western Ontario. Participants reported speaking English (L2) 20–90% of the time. Their mean L2 English usage was 56.96%, and their mean L2 AoA was 6.9 years. It is worth noting that for these participants, VPCs do not exist in their L1 (French), thus we controlled for L2 English usage in the first two analyses because it coarsely measures the opportunities that L2 users have to engage with VPCs.

Participants read the same 78 VPCs as Experiment 1 taken from Blais and Gonnerman (2013) embedded into 78 English sentences containing an adjacent or split form of the VPC. Each participant only saw each VPC in one of the two conditions (adjacent or split), but never both. In Analysis 2 and 3 of Experiment 2, we again assessed the role of two linguistic properties of each VPC: frequency and verb-particle co-occurrence strength. In all analyses, COCA frequency of the adjacent verb + particle form served as the metric for frequency, and the LSA cosine values of overlapping verb and particle context served as the metric for co-occurrence strength. A more positive score indicates greater co-occurrence strength between the verb and the particle (more semantically transparent), whereas a more negative score suggests less co-occurrence strength (more semantically opaque).

The data analytic procedures were the same as those in Experiment 1. Here, we conducted three sets of analyses for L2 reading of VPC: Analysis 1 assessed participants’ general preference for particle placement while controlling for L2 English usage, Analysis 2 explored whether participants’ preference for particle placement varied as a function of VPC frequency and co-occurrence strength while controlling for L2 English usage, and Analysis 3 specifically explored the interaction between item-specific properties and L2 English usage.

### Analysis 1: Does Form Modulate L2 VPC Reading?

We computed an LMM with random intercepts by subjects and items for the core interaction between reading measure (gaze duration vs. second pass reading time) × VP position (adjacent vs. split) × region (VPC vs. post-VPC spillover region). We included percent of current L2 English usage as a covariate in this model because the participants in this sample greatly varied in the extent of their English experience.^3^ Similar to Experiment 1, we predict that if L2 readers generally prefer one VPC form over the other, that the preference will be given to the adjacent form due to decreased processing demands.

We found two significant main effects: reading measure (β = −4.67, *SE* = 0.05, *p* < 0.001) and region (β = −0.60, *SE* = 0.07, *p* < 0.001). These patterns indicate that overall, first pass reading times were longer than second pass reading times, and that reading times were longer at the VPC region compared to the post-VPC spillover region. The effect of region was maintained when we controlled for the difference in length between the VPC and post-VPC spillover regions (β = −0.60, *SE* = 0.07, *p* < 0.001). There were two significant two-way interactions. The first interaction was between reading measure and region (β = −0.55, *SE* = 0.10, *p* < 0.001). Similar to Experiment 1, we computed the difference in reading time at the VPC and post-VPC regions during first and second pass reading. Whereas this difference was numerically larger during first pass reading (287 ms vs. 128 ms), the percent decrease in reading time from VPC to post-VPC spillover region was greater during second (67.1%) than first pass reading (39.6%). Again, this interaction was maintained when we controlled for the difference in length between the two regions (β = −0.55, *SE* = 0.10, *p* < 0.001).

**FIGURE 4:**
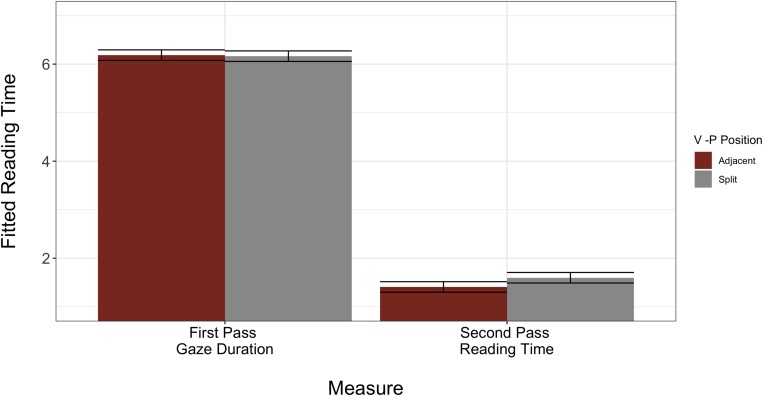
Model fitted L2 reading times for core model interaction between reading measure × VP position. Error bars indicate plus/minus one standard error of the mean.

The second and more critical interaction was between reading measure and VP position (β = −0.21, *SE* = 0.10, *p* = 0.04). To better understand the nature of this interaction, we conducted follow up LMMs on subsets of only first pass reading and only second pass reading. We decided to divide the dataset in this manner because it controls for variability in length of both regions (VPC and post-VPC spillover) by comparing the adjacent and split forms of each item set against each other. From these models, we discovered that during first pass reading, L2 readers display no preference for adjacent or split form, as demonstrated by similar reading times across the conditions (β = −0.006, *SE* = 0.03, *p* = 0.81). However, second pass reading for split items is greater (i.e., slower) than second pass reading time for adjacent items (β = 0.17, *SE* = 0.10, *p* = 0.09). Though this follow-up effect did not reach significance, its direction indicates that L2 readers allocated numerically more reading time to the split form of all VPCs during rereading, which may suggest additional processing costs with this form (Figure 4, with full model outputs available in Table 4). Once again, the model residuals displayed non-normal distribution.

**TABLE 4:** Experiment 2, Analysis 1 core model outputs.

**Fixed effects**	***b***	***SE***	***t-value***	***p-value***
(Intercept)	6.17	0.14	45.54	< 0.001^*^
Measure	–4.67	0.05	–91.23	< 0.001^*^
Region	–0.60	0.07	–8.25	< 0.001^*^
VP Position	0.02	0.07	0.29	0.77
L2 Usage	0.00	0.19	–0.02	0.98
Measure × Region	–0.55	0.10	–5.39	< 0.001^*^
Measure × VP Position	–0.21	0.10	–2.06	0.04^*^
Region × VP Position	–0.05	0.15	–0.36	0.72
Measure × Region × VP Position	–0.16	0.20	–0.77	0.44

**Random effects**	**Variance**			

Item (Intercept)	0.03			
Participant (Intercept)	0.24			

^*^p < 0.05.

lmer[Log RT ∼ Measure^*^Region^*^Position + Scaled L2 Usage + (1 | Subject) + (1 | item), vp_L2].

Lastly, we considered the distribution of frequency for our VPC items. One item in particular (*find out*) demonstrated extremely high frequency in comparison to the other items (see Figure 1). We reran this model without the high frequency item and discovered that the significant effect of VP position from Analysis 1 did not hold. In other words, L2 preference for adjacent VPCs overall, as demonstrated by shorter reading times, was not maintained with the removal of this item.

### Analysis 2: Do Co-occurrence Strength and Frequency Modulate L2 VPC Reading?

In Analysis 1, we found an overall trend for L2 readers to display longer second pass reading time for split VPCs at the VPC region. Next, we assessed whether specific types of items impacted preference for particle placement. Past work indicates that L2 readers make use of item-level differences in semantic transparency during self-paced reading, thus the aim of this analysis was to determine whether these preferences extend to a natural reading paradigm. We computed a series of LMMs with random intercepts by subjects and items for the item-specific models. We fit an interaction between VP position (adjacent vs. split) × scaled verb particle frequency × scaled co-occurrence strength. This interaction was tested for each region and measure individually, thus resulting in four models (gaze duration at the VPC, gaze duration at the Post-VPC spillover, second pass reading time at the VPC, and second pass reading time at the Post-VPC spillover). Of these models, there were no significant item-specific interactions with VP position; however, we detected a near significant main effect of VP position in gaze duration at the VPC region across all items (β = 0.07, *SE* = 0.04, *p* = 0.06; full model outputs are available in Supplementary Materials).^4^ Taken together, the data do not support the interpretation that L2 preference for adjacent VPCs varies as a function of item differences in frequency or semantic co-occurrence strength.

### Analysis 3: Does L2 English Usage Modulate L2 VPC Reading?

Analysis 2 indicated that L2 readers processed all VPC items similarly. In Analysis 3, we investigated whether individual differences in English experience (operationally defined as general percentage of L2 English usage) interacted with processing of item-specific properties. Past work has demonstrated that among English–French bilinguals tested in Montreal, extent of knowledge with VPCs predicts reading behavior (Herbay et al., 2018). While we do not have an explicit measure of VPC knowledge for our sample, we used percentage of L2 English usage as an approximation of the opportunities that L2 users have to use or be exposed to VPCs, given that VPCs are only present in English and not in French. We predicted that greater L2 English usage aligns with greater VPC knowledge, and thus, as found in Herbay, Baum, and Gonnerman, these readers will demonstrate a preference for adjacent particle placement for opaque VPCs (as found for L1 readers), whereas bilinguals with less L2 English usage may not demonstrate this sensitivity to likely lexicalized VPCs.

We computed a series of LMMs with random intercepts by subjects and items for the item-specific models. We fit an interaction between VP position (adjacent vs. split) × scaled verb particle frequency × scaled co-occurrence strength × scaled L2 usage. This interaction was tested for each region and measure individually, thus resulting in four models (gaze duration at the VPC, gaze duration at the Post-VPC spillover, second pass reading time at the VPC, and second pass reading time at the Post-VPC spillover). Of these models, there were no significant item-specific or participant-specific interactions with VP position; however, we detected two borderline effects. The first indicated a main effect of VP position during gaze duration at the VPC region, much like Analysis 2 (β = 0.09, *SE* = 0.05, *p* = 0.05). The second indicated a near significant interaction between VP position and L2 English Usage during second pass reading at the post-VPC spillover region (β = 0.51, *SE* = 0.28, *p* = 0.07).^5^ Taken together, though no interactions involving VP position reaching statistical significance, there is a trend for L2 English users with low English usage to undergo additional processing costs when reading split VPCs in general, regardless of item characteristics.

## Experiment 2 Discussion

Experiment 2 examined L2 reading of VPCs in a different sample of bilingual adults that greatly varied in their L2 English usage (range: 20–90%). Analysis 1 examined general preference for particle placement while controlling for L2 English usage, given the large variability in this measure. There were two findings from the first analysis. First, a significant interaction between reading measure and region indicated that despite VP position, L2 readers go back to the target VPC region during second pass reading for longer periods of time compared to the post-VPC spillover region. One interpretation of this result may be that L2 readers generally need extra time to process VPCs specifically, but this extra processing time is not necessary for the simpler, non-figurative parts of the sentence (i.e., post-VPC spillover region). Indeed, this effect does not depend on the difference in length between the regions because the effect was maintained when we controlled for number of characters in each region. Thus, it is not that L2 readers globally reread parts of the sentence, but rather that their refixation times were strategic and likely suggestive of more challenging or less familiar constructs, such as all VPCs generally.

The second and more critical finding involved an interaction between reading measure and VP position for all items. This indicated that second pass reading times, regardless of region, were longer for split vs. adjacent forms. In other words, L2 readers needed more time to process split VPCs compared to adjacent forms during rereading, suggesting a preference for the adjacent form. We did not detect this effect in Analysis 2 or Analysis 3, which suggests that this effect may be weak and could rely on the totality of the data to be detected.^6^ Thus, we find a trend for L2 readers to prefer adjacent VPCs overall during second pass reading, a pattern that is not affected by item-level differences in frequency or semantic co-occurrence strength.

Why might L2 readers prefer adjacent VPCs during rereading? Given the greater frequency of VPCs in the adjacent form vs. split form in the linguistic environment (Lohse et al., 2004), L2 readers may be more familiar with this form. Related to this point, we discovered that the L2 preference for adjacent VPCs overall, as demonstrated by shorter reading times, was not maintained with the removal of one highly frequent item. This provides additional evidence that L2 preference for VPC particle placement is heavily influenced by global frequency distributions in the linguistic environment. As a result, we would expect that more frequent VPCs would be read more quickly than less frequent VPCs.

Analysis 2 assessed whether global preference for adjacent vs. split particle placement was modulated by each item’s linguistic dimensions. Contrary to our expectations, these models did not reveal any significant interactions with VP position, thus we did not find evidence that L2 reading of VPCs is impacted by item differences in frequency and co-occurrence strength. Furthermore, the failure to detect an effect of semantic co-occurrence strength was again unexpected, given past work suggesting that L2 readers have more difficulty with VPCs that vary in how idiomatic they seem (Liao and Fukuya, 2004; Blais and Gonnerman, 2013). However, it is important to note critical design differences in past work and the present study, the most egregious difference being how “difficulty with VPCs” was measured. Past work overwhelmingly has relied on paradigms useful for language learning (e.g., multiple choice tests, production tasks; Dagut and Laufer, 1985; Laufer and Eliasson, 1993; Liao and Fukuya, 2004) or self-paced reading (e.g., Blais and Gonnerman, 2013; Herbay et al., 2018), whereas the present work assesses processing through a more naturalistic reading paradigm using eye-tracking. It is possible that although L2 users avoid opaque VPCs and prefer single word synonyms during actual use of the L2, they do not demonstrate implicit processing differences as a function of linguistic dimension (for a similar discussion see Paulmann et al., 2015). This relates to a common idea in the avoidance literature that perhaps avoidance behavior stems from *perceived* difficulty with less familiar constructs (Sjoholm, 1995).

Indeed, it is quite possible that the comparable reading times between opaque/transparent and frequent/infrequent VPCs arose because L2 readers are superficially skimming past the difficult VPCs without accessing their meaning; however, L2 readers performed quite accurately on the comprehension questions scattered throughout the experiment (89.6% accurate, compared to 90.3% for L1 readers), which suggests that they are generally reading the sentences for comprehension. Other key differences between past work and the present study include the proficiency or L2 usage of the L2 sample (our sample was quite varied in L2 usage; however, they all resided in the linguistically unique environment of Montreal where both French and English are frequently used in daily life) and L1-L2 similarity. As discussed, VPCs are present in English but not in French. This means that L2 readers have limited opportunities to experience VPCs, which is constrained by the extent to which they use their L2.

Analysis 3 examined whether individual differences in L2 English usage predicted form preference overall and whether these experiences differentially affected VPCs that varied in frequency and semantic co-occurrence strength. Although we predicted that L2 English readers with greater English usage, who presumably have more opportunities to engage with VPCs, would demonstrate a preference for adjacent particle placement for opaque VPCs (similar to L1 readers), the analysis revealed a near significant difference for L2 English readers with low English usage, though this effect failed to reach the threshold for significance (see Footnote 5). One potential reason we failed to detect a robust difference among readers might be the proxy measure used for VPC knowledge (general percentage L2 usage). It is possible that this coarse variable did not capture the subtle individual differences in VPC knowledge. Thus, one avenue of future research would be to directly assess how knowledge of specific VPC influences natural reading patterns.

## General Discussion

We investigated bilingual reading of form-flexible idioms in verb-particle constructions. Specifically, we examined preference for particle placement in general across L1 and L2 reading. We also assessed whether particle placement varied as a function of frequency and co-occurrence strength. Lastly, we examined whether these patterns were influenced by L2 English usage during L2 reading. There were several key findings pertaining to L1 and L2 reading of particle placement preference and the linguistic constraints under which these preferences emerge.

### L1 Reading of VPCs Is Modulated by Both Frequency and Semantic Co-occurrence Strength

The core model (Analysis 1) did not detect a processing difference between adjacent and split items during L1 reading. Thus, we next assessed whether linguistic constraints—frequency and co-occurrence strength—modulated reading of adjacent and split VPCs. Here, we found that L1 readers demonstrated sensitivity to particle placement for certain types of items. Specifically, VPCs high in frequency and low in co-occurrence strength (e.g., *cut back* or *add up*) were preferred in the adjacent form. In fact, first pass reading time for these items was the fastest across all items (see Figure 3). Of interest, items that were low in semantic co-occurrence strength *but also* low in frequency were not conferred a processing advantage in the adjacent form. In fact, as illustrated in Figure 3, the reading time for split forms of these items did not change, but reading time for the adjacent form slowed down.

One way to understand these results is to consider that low co-occurrence items are highly likely to be lexicalized. In other words, the VPC is likely stored in the lexicon as one unit, as opposed to being compositionally built. These lexicalized VPCs that are also highly frequent are retrieved the most quickly from the lexicon. Thus, it is possible that L1 English readers, who presumably have more exposure to VPCs than L2 English readers, have accumulated a broad range of familiarity with VPCs and are more experienced to extract subtle linguistic differences between items. Items that are easy to directly retrieve from the lexicon (low semantic co-occurrence and high frequency) will be processed more quickly in the adjacent form compared to items that do not benefit from the interactional boost of being semantically opaque *and* highly frequent. Additional evidence for this explanation can be drawn from a visual analysis of Figure 3, where low frequency, opaque VPCs in the adjacent form actually slow down reading, presumably because the lexicalized meaning cannot be quickly retrieved.

The L1 reading patterns align to some degree with past findings from the monolingual literature. In Gonnerman and Hayes (2005), monolinguals demonstrated a preference for adjacent particle placement when the VPC was opaque. However, this past work also revealed a general monolingual preference for adjacent VPCs overall, which we did not detect with bilinguals reading in their L1. This divergence is similar to the mismatch between current and early findings of classic idioms, more specifically related to the facilitative vs. interfering role of semantic transparency on reading (e.g., Titone et al., 2019). Indeed, the eye-tracking data from the present work indicates that L1 preferences emerge early on, during first pass reading, as opposed to emerging upon rereading. Thus, the present work corroborates and adds to these findings by assessing the role of frequency in modulating the effects of semantic transparency.

Contrary to our expectations, the L2 readers did not demonstrate sensitivity to item-level differences, as L1 readers. Instead, these readers demonstrated a trend for more general processing preference for adjacent VPCs during rereading, irrespective of frequency and semantic co-occurrence strength.

### L2 Reading of VPCs Is Likely Modulated by Form

In the core model (Analysis 1), L2 readers’ reading times for adjacent and split VPC forms diverged. Specifically, we found significantly longer second pass reading times for split particles compared to adjacent particles among L2 readers. This result suggests that during first pass reading, L2 readers do not demonstrate strong preference for adjacent vs. split particle placement. However, upon refixating and rereading, there is a trend for L2 readers to linger on split particle forms but not adjacent particle forms. Thus, we find evidence that only L2 readers demonstrate different reading patterns for adjacent vs. split VPCs irrespective of item differences in frequency or semantic co-occurrence strength.

We propose that this additional reading time reflects a processing cost for the less preferred split particle placement. Past corpus analyses of VPCs have revealed that VPCs in English are more commonly found in the adjacent form (Lohse et al., 2004). Our own corpus analysis of the VPCs in the present work corroborates this conclusion. Thus, it is possible that L2 users of English are simply more familiar with the adjacent form than the split form of any VPC. However, it is unlikely that this is the final story, because as we found in Analysis 2, L2 readers do not change their reading patterns for more or less frequent VPCs.

An alternative explanation for this pattern of results could stem from an L2 shallow processing perspective (Clahsen and Felser, 2006). Take the VPC *eat up*, for example. Our results indicate that if an L2 reader encountered this expression in the split form (*eat the candy up*), they will show longer second pass reading times compared to the adjacent form (*eat up the candy*). Presumably, without the particle the expression is still fully understandable (*eat the candy*), but once the particle is read, one may think that there needs to be a reevaluation (i.e., longer second pass reading times) because otherwise the particle is vestigial. The tendency to reevaluate may be heighted for an L2 reader who may struggle with acute differences in the L2. There was some evidence for this pattern from Analysis 3, which revealed a near significant interaction between VP position and L2 English usage. Indeed, it may not matter whether the presence of the split particle actually changes the meaning of the verb or not (i.e., semantic transparency). What may be more impactful to the L2 reader is the perception that a dangling particle must be resolved (e.g., see de Angelis, 2005 for discussion of L2 preference for native vs. non-native function words).

However, the results for L2 reading diverge from past findings, which typically show that L2 readers are also sensitive to semantic differences among VPCs (e.g., Blais and Gonnerman, 2013; Herbay et al., 2018). It is possible that L2 readers demonstrate explicit sensitivity (and even implicit sensitivity as measured by a masked prime task) to differences in semantic transparency among VPCs; however, to our knowledge there has not been a naturalistic investigation of solely L2 VPC reading using eye tracking. Cieślicka et al. (2014) used eye-tracking to contrast L1 vs. L2 idiomatic reading with materials that included VPCs among other forms of idiomatic language, but they did not analyze reading of VPCs specifically, nor did they assess the same item-level characteristics. They found that for all idiomatic language (including VPCs and various forms of classic idioms) bilinguals were faster at recognizing idioms in the dominant vs. non-dominant language. In other words, bilingual eye fixations of idioms were more rapid in a context that is comparable to the L1.

Similarly, past investigations on the role of frequency in predicting multi-word expression reading patterns have been limited. For example, Siyanova-Chanturia et al. (2011) used eye-tracking to assess whether the frequency of collocated, binomial phrases (e.g., *bride and groom)* predicted reading of the expression as compared to the reverse of the expression (e.g., *groom and bride*). They found that both L1 and L2 readers demonstrated sensitivity to these frequency differences for multi-word expressions. Here, we observed sensitivity to frequency among L1 readers only; however, L2 reading patterns were globally aligned with the frequency of particle placement forms in the linguistic environment (i.e., adjacent VPCs are more common than split).

We also make note that the failure to detect L2 processing constraints as a function of item characteristics may be due to a lack of statistical power.^7^ The L2 reading group had fewer participants, who also varied more than the L1 group on multiple individual difference measures, including L2 usage. Furthermore, as discussed earlier, the inclusion of zero second pass reading times led to a deviation from normality for the model residuals, which relates to loss of power and a potential increase in the likelihood of a Type II error. For these reasons, it is possible that our failure to detect differences in how linguistic properties affect L2 reading arose from a lack of power (although the same issues of model non-normality were similarly true of our analyses of L1 readers in Experiment 1). To remedy this limitation, we encourage future research to work with larger sample sizes and consider alternatives to zero-included second pass reading times (see Vasishth et al., 2012 for more discussion on this).

### L2 English Usage May Modulate VPC Reading

Given that L2 readers only demonstrate preference for adjacent VPC forms during rereading, we affirmatively tested the interaction between L2 English usage, the linguistic constraints, and particle placement preference. This model only returned a near significant interaction between VP position and L2 English usage during second pass reading of the post-VPC spillover region (see Footnotes 4–6), which suggested that processing costs attributed to the split form may be exacerbated for L2 users who are less familiar with English. Despite this weak effect, there was no evidence that L2 English modulated preference for VPCs as a function of linguistic characteristics (i.e., no interaction with frequency or semantic co-occurrence strength). Again, these findings differ from the results of Herbay et al. (2018) who found that L2 readers more familiar with VPCs prefer opaque VPCs in the adjacent form. As discussed, it is possible that our coarse, proxy measure of VPC familiarity was not precise enough to capture true differences in individuals’ knowledge of VPCs. Thus, it may not be possible to directly compare these two findings. Moreover, in Herbay et al. (2018), individual differences in working memory capacity were also involved with differences in VPC reading patterns. Additionally, their participants performed fairly high on a written cloze probability task that assessed L2 proficiency (mean score = 24.3 out of 30; standard deviation = 4.5), whereas our sample greatly varied in their L2 usage (20–90%). Lastly, these participants also took part in other tasks that are reported in Blais and Gonnerman (2013), including an explicit VPC semantic rating task. It is possible that those participants were made aware that the goal of the experiment was to assess VPC knowledge, in which case their attention to the VPC reading task may have been affected by that understanding. For these reasons, our novel results add nuance to pre-existing data related to L2 reading of VPCs.

Taken together, these results suggest that there are measurable differences in how L1 and L2 readers process form flexible idiomatic language (VPCs). L1 readers rely on a multitude of linguistic constraints, such as frequency and co-occurrence strength, that shape their preference for VPC particle placement. L2 readers, on the other hand, do not show this same strategy, instead displaying a global preference for the adjacent form during rereading. There is some evidence suggesting that this preference may vary as a function of self-reported L2 English usage; however, this effect was not robust. This suggests that L2 readers may have a vague familiarity with VPCs from the linguistic environment in general that pushes them in favor of the adjacent form. However, this familiarity may not be strongly influenced by the frequency of individual items, as it is for L1 readers. Lastly, there is some evidence that the time course for the emergence of these preferences during reading also differs. L1 readers demonstrate item preference during first pass reading, whereas overall form preference emerges during second pass reading for L2 users. This hints at a possible automaticity among L1 readers to hone in on subtle semantic and frequency cues during reading, whereas L2 readers more intentionally allocate additional processing resources to resolve the potentially more difficult split form VPC. Furthermore, any processing differences between adjacent and split VPC forms detected in the present work are likely on the weaker side for what is possible given that the noun phrase across all items consisted of two words (determiner + noun). Past work has demonstrated that processing difficulties for split VPCs are exacerbated by longer intervening noun phrases (e.g., *The girl will eat the super delicious and scrumptious candy up all at once*.), even among L2 readers (Herbay et al., 2018). Thus, we expect that if the present items consisted of longer or more variable noun phrase lengths, the weak preference for adjacent VPC forms detected here would likely be strengthened.

It is important to note that our regions of interest were quite large. In most sentences, the VPC and Post-VPC regions comprised of about half the sentence (verb + particle + noun phrase). We analyzed both regions because processing does not stop once the eyes move outside the region of the VPC itself. In fact, it is likely that any differential effects within a region could be continued processing from the immediately preceding region (Mitchell, 1984). L2 preference for the adjacent particle did not vary as a function of region, but L1 reading did. L1 readers demonstrate a preference for the adjacent form immediately: during first pass reading of the VPC region and not at the spillover region. This provides further evidence that fine-grained sensitivity to different VPC forms is a more effortful process for L2 readers, whereas L1 readers demonstrate clearer automaticity in their VPC reading strategy.

Moreover, the length of each region varied across items, which may have added error variance to these analyses. The presence of this noise may have contributed to our failure to detect significant effects, specifically among L2 readers who may be more sensitive to length than L1 readers.^8^ To address this point, we conducted a simple follow-up analysis to Experiment 2, where character length of the VPC and post-VPC spillover regions were added as covariates to Analysis 1–3 for L2 reading. Indeed, reducing this variance brought the *p*-value for the main effect of VP position on gaze duration at the VPC region closer to the significance threshold (Analysis 2: β = 0.07, *SE* = 0.04, *p* = 0.05; Analysis 3: β = 0.10, *SE* = 0.05, *p* = 0.05). The results of Analysis 1 did not change. No further interactions involving VP position were detected. Approaching the follow-up analysis in this manner suggests that the failure to initially detect a main effect of VP position in Analysis 2 and 3 might have been related to variance in region length across items.

Finally, these results are interesting in light of what we know about generally less structurally flexible idioms (e.g., *kick the bucket*). Our results from VPCs mirror classic findings in the idiom literature: processing is fast to the extent that a particular construction is highly frequent at the word-form level, and that construction is encountered in that highly entrenched form. For example, in a cross-modal priming study L1 comprehenders heard sentences containing idioms and made lexical decisions to targets relating to these idioms’ figurative meanings (Titone and Libben, 2014). The authors found that *increased* phrase frequency (but not semantic transparency) *increased* figurative priming at very short prime-target intervals. In contrast, at later prime-target intervals, *increased* semantic transparency *decreased* figurative priming. This suggests that factors leading to greater lexicalization of phrasal idioms (familiarity) were facilitative, however, factors leading to greater semantic analysis (transparency) led to slower figurative processing. Similarly, in a recent eye-tracking study L1 readers naturally read idioms embedded in sentences (Titone et al., 2019). Here, the authors found that all idioms as a class were read more quickly than matched literal phrases for early reading measures (idiom gaze duration). However, for later reading measures (idiom total reading time), *increased* frequency *speeded* reading times, however, *increased* semantic transparency *slowed* reading times.

Taken in concert with this idiom work, the VPC results presented here converge on the idea that L1 readers prefer to treat overlearned sequences in a lexicalized, formulaic way. When they do, this speeds the early stages of language processing. These findings also converge on the idea that increased semantic transparency might actually slow comprehension for formulaic language (in direct contrast with classic accounts of compositionality; e.g., Gibbs et al., 1989), because high semantic transparency likely encourages readers to adopt a more deliberative, metaphorical, or compositional mode of comprehension. Accordingly, when readers encounter idioms, metaphors, or VPCs in their L1, where the word parts relate to the phrasal whole, they analyze the particular combination of semantics in the moment. Likely, they do so in a manner that leads to a slower appreciation of the figurative meanings of these sequences (see also Wray, 1999, for a similar account of formulaic language processing).

## Conclusion

The present study demonstrated that VPC reading in L1 and L2 differed for English–French bilingual adults. L1 readers exhibited nuanced form preferences for early measures of reading; they demonstrated a preference for adjacent particles for items that are promoted by direct retrieval, specifically, highly transparent and frequent VPCs. In contrast, L2 readers displayed more general preference for adjacent particles, as demonstrated by greater second pass reading time for all split items. This preference is likely more effortful and may depend on the reader’s familiarity with VPCs. Whether this pattern of results during natural reading extends to alternative but similar types of figurative language, such as idiomatic expressions, is a question that we are currently pursuing.

## Ethics Statement

This study was carried out in accordance with the recommendations of McGill University’s Research Ethics Board with written informed consent from all subjects. All subjects gave written informed consent in accordance with the Declaration of Helsinki.

## Author Contributions

LG created the items and DT modified them. LG, VW, and DT designed the experiments. MT, VW, DF, DJ, and DT contributed to the data acquisition. MT and DT processed and analyzed the data. MT wrote the final manuscript and all other authors critically revised it. All authors listed have made a substantial and direct contribution to the work and approved it for publication.

## Conflict of Interest Statement

The authors declare that the research was conducted in the absence of any commercial or financial relationships that could be construed as a potential conflict of interest.

## Footnotes

1 http://lsa.colorado.edu/; though note that this web page is no longer active as of July 21, 2019.

2 https://osf.io/vr7cy/

3 Note that omitting this covariate term did not impact the results of this model.

4 To investigate the nature of this near significant effect, which was not previously detected in Analysis 1, we calculated a follow- up model for gaze duration at the VPC region with only VP position as a predictor (plus L2 English usage as a covariate). This model did not return a significant effect of VP position (β = 0.04, *SE* = 0.03, *p* = 0.20), but a visual analysis suggested that gaze duration for split vs. adjacent items was slightly faster at the VPC region.

5 To investigate the nature of this near significant interaction, we split the L2 sample in half based on the median value of L2 English usage. Then, we calculated a model for second pass reading at the post-VPC spillover region with only VP position as a predictor. Among L2 English users with high English usage, there was no significant effect of VP position (β = −0.08, *SE* = 0.16, *p* = 0.61). Among L2 English users with low English usage, there was a significant main effect of VP position (β = −0.47, *SE* = 0.16, *p* < 0.01). This effect indicates that during second pass reading of the post-VP spillover region, L2 English readers with low English usage spend significantly more time reading the split vs. adjacent VPC form (full model outputs are available in Supplementary Materials).

6 A potential third finding relates to several near significant or borderline effects that were detected from Analysis 2 and Analysis 3. Namely, there was evidence to suggest that L2 readers may have noticed differences between adjacent and split VPCs during first pass reading. In Analysis 2, a follow-up analysis provided some evidence that gaze duration at the VPC region was shorter for split vs. adjacent VPCs. In contrast, Analysis 3 suggested that second pass reading of split VPCs was slower than that of adjacent VPCs in the post-VPC spillover region, but only for L2 English readers with low English usage (similar to Analysis 1). Taken together and interpreted cautiously, it is possible that when L2 readers first encounter a split VPC, they read the isolated verb easily. Then, once they read the particle at the end of the VPC region, they undergo processing difficulties, as demonstrated by longer fixation times in the post-VPC spillover region for split VPCs. Moreover, some evidence suggests that this processing cost is exacerbated for L2 English readers who are likely less familiar with VPCs.

7 We assessed whether the pattern of results detected across Experiments 1 and 2 varied as a function of different sample sizes (56 in E1; 27 in E2). We randomly selected 27 participants from E1 and reran all analyses on that subset. The critical three-way interaction from Analysis 2 was maintained with this subset.

8 Indeed, controlling for character length during L1 reading (Experiment 1) did not affect the critical interaction between VP position × frequency × co-occurrence strength during first pass reading at the VPC region (Analysis 2). The model in Analysis 1 including this covariate failed to converge.
