# Supplementary material for: Figuring Out How Verb-Particle Constructions Are Understood During L1 and L2 Reading
Source: Front Psychol. 2019 Jul 31;10:1733. doi: 10.3389/fpsyg.2019.01733 (PMC6684791; doi:10.3389/fpsyg.2019.01733)
Supplement: Supplementary file 1 [file Table_1.DOCX]

Table 6A: Experiment 2, Analysis 2 Item-Specific Model Outputs at the VPC Region

| **First Pass Gaze Duration** | | | | |  |
| --- | --- | --- | --- | --- | --- |
| Fixed Effects | *b* | *SE* | *t-value* | *p-value* |  |
| (Intercept) | 6.41 | 0.06 | 99.59 | <0.001* |  |
| VP Position | 0.07 | 0.04 | 1.92 | 0.06 | . |
| Frequency (scaled) | 0.08 | 0.11 | 0.70 | 0.49 |  |
| Co-occurrence Strength (scaled) | -0.05 | 0.04 | -1.18 | 0.24 |  |
| L2 Usage | -0.10 | 0.09 | -1.18 | 0.25 |  |
| VP Position x Frequency (scaled) | 0.25 | 0.19 | 1.37 | 0.17 |  |
| VP Position x Co-occurrence Strength (scaled) | 0.05 | 0.07 | 0.75 | 0.45 |  |
| Frequency (scaled) x Co-occurrence Strength (scaled) | -0.12 | 0.17 | -0.69 | 0.49 |  |
| VP Position x Frequency (scaled) x Co-occurrence Strength (scaled) | -0.38 | 0.28 | -1.35 | 0.18 |  |

| Random Effects | | Variance | | | |  |
| --- | --- | --- | --- | --- | --- | --- |
| Item (Intercept)  Participant (Intercept) | | 0.01  0.05 | | | |  |
| **Second Pass Reading Time** | | | | | |  |
| Fixed Effects | *b* | | *SE* | *t-value* | *p-value* |  |
| (Intercept) | 2.09 | | 0.36 | 5.81 | < 0.001* |  |
| VP Position | -0.06 | | 0.19 | -0.34 | 0.74 |  |
| Frequency (scaled) | -0.94 | | 0.64 | -1.47 | 0.15 |  |
| Co-occurrence Strength (scaled) | 0.28 | | 0.22 | 1.29 | 0.20 |  |
| L2 Usage | 0.11 | | 0.50 | 0.23 | 0.82 |  |
| VP Position x Frequency (scaled) | -0.55 | | 1.01 | -0.54 | 0.59 |  |
| VP Position x Co-occurrence Strength (scaled) | -0.17 | | 0.36 | -0.49 | 0.63 |  |
| Frequency (scaled) x Co-occurrence Strength (scaled) | 1.23 | | 0.97 | 1.28 | 0.21 |  |
| VP Position x Frequency (scaled) x Co-occurrence Strength (scaled) | -0.03 | | 1.50 | -0.02 | 0.98 |  |
| Random Effects | Variance | | | | |  |
| Item (Intercept)  Participant (Intercept) | 0.26  1.60 | | | | |  |

* p < 0.05

*Note.* Gaze Duration: lmer(Log RT ~ position*scaled frequency*scaled co-occurrence + scaled L2 Usage + (1 | Subject) + (1 | item), L2_VP_GD). Second Pass: lmer(Log RT ~ position*scaled frequency*scaled co-occurrence + scaled L2 Usage + (1 | Subject) + (1 | item), L2_VP_SP)

Table 6B: Experiment 2, Analysis 2 Item-Specific Model Outputs at the Post-VPC Region

| **First Pass Gaze Duration** | | | | | |  | | |  |
| --- | --- | --- | --- | --- | --- | --- | --- | --- | --- |
| Fixed Effects | *b* | *SE* | *t-value* | *p-value* | |  | | |  |
| (Intercept) | 5.79 | 0.09 | 61.90 | < 0.001* | |  | | |  |
| VP Position | -0.02 | 0.04 | -0.43 | 0.66 | |  | | |  |
| Frequency (scaled) | -0.06 | 0.21 | -0.27 | 0.79 | |  | | |  |
| Co-occurrence Strength (scaled) | -0.04 | 0.07 | -0.60 | 0.56 | |  | | |  |
| L2 Usage | -0.06 | 0.12 | -0.48 | 0.63 | |  | | |  |
| VP Position x Frequency (scaled) | 0.21 | 0.20 | 1.04 | 0.30 | |  | | |  |
| VP Position x Co-occurrence Strength (scaled) | -0.06 | 0.07 | -0.82 | 0.41 | |  | | |  |
| Frequency (scaled) x Co-occurrence Strength (scaled) | 0.13 | 0.32 | 0.41 | 0.69 | |  | | |  |
| VP Position x Frequency (scaled) x Co-occurrence Strength (scaled) | -0.19 | 0.31 | -0.61 | 0.54 | |  | | |  |
| Random Effects | Variance | | | | | |  |  | |
| Item (Intercept)  Participant (Intercept) | 0.06  0.10 | | | | | |  |  | |
| **Second Pass Reading Time** | | | | | | |  |  | |
| Fixed Effects | *b* | *SE* | *t-value* | | *p-value* | |  |  | |
| (Intercept) | 0.98 | 0.21 | 4.69 | | < 0.001* | |  |  | |
| VP Position | -0.24 | 0.15 | -1.66 | | 0.10 | |  |  | |
| Frequency (scaled) | 0.23 | 0.47 | 0.49 | | 0.62 | |  |  | |
| Co-occurrence Strength (scaled) | 0.04 | 0.16 | 0.27 | | 0.79 | |  |  | |
| L2 Usage | 0.03 | 0.28 | 0.12 | | 0.91 | |  |  | |
| VP Position x Frequency (scaled) | -0.02 | 0.78 | -0.02 | | 0.98 | |  |  | |
| VP Position x Co-occurrence Strength (scaled) | -0.10 | 0.27 | -0.35 | | 0.73 | |  |  | |
| Frequency (scaled) x Co-occurrence Strength (scaled) | -0.31 | 0.71 | -0.43 | | 0.67 | |  |  | |
| VP Position x Frequency (scaled) x Co-occurrence Strength (scaled) | -0.60 | 1.17 | -0.52 | | 0.61 | |  |  | |
| Random Effects | Variance | | | | | |  |  | |
| Item (Intercept)  Participant (Intercept) | 0.12  0.48 | | | | | |  |  | |

* p < 0.05

*Note.* Gaze Duration: lmer(Log RT ~ position*scaled frequency*scaled co-occurrence + scaled L2 Usage + (1 | Subject) + (1 | item), L2_Spill_GD). Second Pass: lmer(Log RT ~ position*scaled frequency*scaled co-occurrence + scaled L2 Usage + (1 | Subject) + (1 | item), L2_Spill_SP)

Table 7A: Experiment 2, Analysis 3 Item-Specific & L2 Usage Model Outputs at the VPC Region

| **First Pass Gaze Duration** | | | | | |  |  |  |
| --- | --- | --- | --- | --- | --- | --- | --- | --- |
| Fixed Effects | *b* | *SE* | *t-value* | *p-value* | |  |  |  |
| (Intercept) | 6.41 | 0.06 | 98.81 | < 0.001* | |  |  |  |
| VP Position | 0.09 | 0.05 | 1.94 | 0.05 | |  |  |  |
| Frequency (scaled) | 0.15 | 0.14 | 1.03 | 0.31 | |  |  |  |
| Co-occurrence Strength (scaled) | -0.07 | 0.05 | -1.45 | 0.15 | |  |  |  |
| L2 Usage | -0.11 | 0.09 | -1.18 | 0.25 | |  |  |  |
| VP Position x Frequency (scaled) | 0.24 | 0.25 | 0.97 | 0.33 | |  |  |  |
| VP Position x Co-occurrence Strength (scaled) | 0.05 | 0.09 | 0.55 | 0.58 | |  |  |  |
| Frequency (scaled) x Co-occurrence Strength (scaled) | -0.12 | 0.22 | -0.54 | 0.59 | |  |  |  |
| VP Position x L2 Usage | 0.05 | 0.07 | 0.79 | 0.43 | |  |  |  |
| Frequency (scaled) x L2 Usage | 0.14 | 0.17 | 0.81 | 0.42 | |  |  |  |
| Co-occurrence Strength (scaled) x L2 Usage | -0.05 | 0.06 | -0.86 | 0.39 | |  |  |  |
| VP Position x Frequency (scaled) x Co-occurrence Strength (scaled) | -0.35 | 0.38 | -0.92 | 0.36 | |  |  |  |
| VP Position x Frequency (scaled) x L2 Usage | -0.01 | 0.35 | -0.04 | 0.97 | |  |  |  |
| VP Position x Co-occurrence Strength (scaled) x L2 Usage | -0.01 | 0.13 | -0.04 | 0.96 | |  |  |  |
| Frequency (scaled) x Co-occurrence Strength (scaled) x L2 Usage | 0.00 | 0.27 | -0.01 | 0.99 | |  |  |  |
| VP Position x Frequency (scaled) x Co-occurrence Strength (scaled) x L2 Usage | 0.06 | 0.53 | 0.12 | 0.90 | |  |  |  |
| Random Effects | Variance | | | |  | | |  |
| Item (Intercept)  Participant (Intercept) | 0.01  0.05 | | | |  | | |  |
| **Second Pass Reading Time** | | | | | |  |  |  |
| Fixed Effects | *b* | *SE* | *t-value* | *p-value* | |  |  |  |
| (Intercept) | 2.12 | 0.36 | 5.86 | < 0.001* | |  |  |  |
| VP Position | 0.08 | 0.26 | 0.30 | 0.76 | |  |  |  |
| Frequency (scaled) | -1.07 | 0.78 | -1.37 | 0.17 | |  |  |  |
| Co-occurrence Strength (scaled) | 0.60 | 0.27 | 2.19 | 0.03 * | |  |  |  |
| L2 Usage | 0.16 | 0.50 | 0.32 | 0.75 | |  |  |  |
| VP Position x Frequency (scaled) | 0.45 | 1.34 | 0.33 | 0.74 | |  |  |  |
| VP Position x Co-occurrence Strength (scaled) | -0.58 | 0.49 | -1.18 | 0.24 | |  |  |  |
| Frequency (scaled) x Co-occurrence Strength (scaled) | 1.04 | 1.19 | 0.87 | 0.39 | |  |  |  |
| VP Position x L2 Usage | 0.31 | 0.36 | 0.85 | 0.39 | |  |  |  |
| Frequency (scaled) x L2 Usage | -0.27 | 0.94 | -0.29 | 0.77 | |  |  |  |
| Co-occurrence Strength (scaled) x L2 Usage | 0.64 | 0.33 | 1.93 | 0.05 | |  |  |  |
| VP Position x Frequency (scaled) x Co-occurrence Strength (scaled) | -1.74 | 2.06 | -0.85 | 0.40 | |  |  |  |
| VP Position x Frequency (scaled) x L2 Usage | 2.00 | 1.90 | 1.05 | 0.29 | |  |  |  |
| VP Position x Co-occurrence Strength (scaled) x L2 Usage | -0.79 | 0.68 | -1.16 | 0.25 | |  |  |  |
| Frequency (scaled) x Co-occurrence Strength (scaled) x L2 Usage | -0.41 | 1.41 | -0.29 | 0.77 | |  |  |  |
| VP Position x Frequency (scaled) x Co-occurrence Strength (scaled) x L2 Usage | -3.49 | 2.87 | -1.22 | 0.22 | |  |  |  |
| Random Effects | Variance | | | | |  |  |  |
| Item (Intercept) | 0.26  1.58 | | | | |  |  |  |
| Participant (Intercept) |  |  |  |  |  |  |  |  |

* p < 0.05

*Note.* Gaze Duration: lmer(Log RT ~ position*scaled frequency*scaled co-occurrence*scaled L2 Usage + (1 | Subject) + (1 | item), L2_VP_GD). Second Pass: lmer(Log RT ~ position*scaled frequency*scaled co-occurrence*scaled L2 Usage + (1 | Subject) + (1 | item), L2_VP_SP)

Table 7B: Experiment 2, Analysis 3 Item-Specific & L2 Usage Model Outputs at the Post-VPC Region

| **First Pass Gaze Duration** | | | | | | |  |
| --- | --- | --- | --- | --- | --- | --- | --- |
| Fixed Effects | *b* | *SE* | *t-value* | *p-value* | | |  |
| (Intercept) | 5.79 | 0.09 | 61.35 | < 0.001* | | |  |
| VP Position | -0.08 | 0.05 | -1.43 | 0.15 | | |  |
| Frequency (scaled) | -0.06 | 0.23 | -0.27 | 0.79 | | |  |
| Co-occurrence Strength (scaled) | -0.10 | 0.08 | -1.28 | 0.20 | | |  |
| L2 Usage | -0.06 | 0.13 | -0.50 | 0.62 | | |  |
| VP Position x Frequency (scaled) | 0.02 | 0.28 | 0.06 | 0.95 | | |  |
| VP Position x Co-occurrence Strength (scaled) | -0.06 | 0.10 | -0.55 | 0.58 | | |  |
| Frequency (scaled) x Co-occurrence Strength (scaled) | 0.15 | 0.35 | 0.42 | 0.68 | | |  |
| VP Position x L2 Usage | -0.12 | 0.07 | -1.65 | 0.10 | | |  |
| Frequency (scaled) x L2 Usage | -0.01 | 0.19 | -0.05 | 0.96 | | |  |
| Co-occurrence Strength (scaled) x L2 Usage | -0.12 | 0.07 | -1.81 | 0.07 | | |  |
| VP Position x Frequency (scaled) x Co-occurrence Strength (scaled) | 0.24 | 0.42 | 0.57 | 0.57 | | |  |
| VP Position x Frequency (scaled) x L2 Usage | -0.40 | 0.39 | -1.03 | 0.30 | | |  |
| VP Position x Co-occurrence Strength (scaled) x L2 Usage | 0.01 | 0.14 | 0.05 | 0.96 | | |  |
| Frequency (scaled) x Co-occurrence Strength (scaled) x L2 Usage | 0.04 | 0.29 | 0.14 | 0.89 | | |  |
| VP Position x Frequency (scaled) x Co-occurrence Strength (scaled) x L2 Usage | 0.87 | 0.58 | 1.49 | 0.14 | | |  |
| Random Effects | Variance | | | | |  | |
| Item (Intercept) | 0.06 | | | | |  | |
| Participant (Intercept) | 0.10 | | | | |  | |
| **Second Pass Reading Time** | | | | | |  | |
| Fixed Effects | *b* | *SE* | *t-value* | | *p-value* |  | |
| (Intercept) | 0.95 | 0.21 | 4.45 | | < 0.001* |  | |
| VP Position | 0.01 | 0.21 | 0.04 | | 0.97 |  | |
| Frequency (scaled) | -0.19 | 0.59 | -0.32 | | 0.75 |  | |
| Co-occurrence Strength (scaled) | 0.07 | 0.20 | 0.34 | | 0.73 |  | |
| L2 Usage | -0.04 | 0.30 | -0.13 | | 0.90 |  | |
| VP Position x Frequency (scaled) | 0.11 | 1.05 | 0.11 | | 0.92 |  | |
| VP Position x Co-occurrence Strength (scaled) | -0.34 | 0.38 | -0.90 | | 0.37 |  | |
| Frequency (scaled) x Co-occurrence Strength (scaled) | 0.23 | 0.89 | 0.26 | | 0.79 |  | |
| VP Position x L2 Usage | 0.51 | 0.28 | 1.84 | | 0.07 |  | |
| Frequency (scaled) x L2 Usage | -0.84 | 0.73 | -1.15 | | 0.25 |  | |
| Co-occurrence Strength (scaled) x L2 Usage | 0.06 | 0.26 | 0.25 | | 0.80 |  | |
| VP Position x Frequency (scaled) x Co-occurrence Strength (scaled) | -0.87 | 1.59 | -0.55 | | 0.59 |  | |
| VP Position x Frequency (scaled) x L2 Usage | 0.25 | 1.47 | 0.17 | | 0.87 |  | |
| VP Position x Co-occurrence Strength (scaled) x L2 Usage | -0.51 | 0.52 | -0.97 | | 0.33 |  | |
| Frequency (scaled) x Co-occurrence Strength (scaled) x L2 Usage | 1.08 | 1.10 | 0.97 | | 0.33 |  | |
| VP Position x Frequency (scaled) x Co-occurrence Strength (scaled) x L2 Usage | -0.53 | 2.22 | -0.24 | | 0.81 |  | |
| Random Effects | Variance | | | | |  | |
| Item (Intercept) | 0.12  0.48 | | | | |  | |
| Participant (Intercept) |  |  |  |  |  |  | |

* p < 0.05

*Note.* Gaze Duration: lmer(Log RT ~ position*scaled frequency*scaled co-occurrence*scaled L2 Usage + (1 | Subject) + (1 | item), L2_Spill_GD). Second Pass: lmer(Log RT ~ position*scaled frequency*scaled co-occurrence*scaled L2 Usage + (1 | Subject) + (1 | item), L2_Spill_SP)
